# A systematic benchmarking pipeline for demand forecasting and inventory-aware supply chain decision support

**DOI:** 10.3389/frai.2026.1936360

**Published:** 2026-10-09

**Authors:** Suleiman Ali Alsaif

**Affiliations:** Computer Department, Deanship of Preparatory Year and Supporting Studies, Imam Abdulrahman Bin Faisal University, Dammam, Saudi Arabia

**Keywords:** deep learning, demand prediction, inventory optimization, machine learning, retail analytics, sales forecasting

## Abstract

Demand forecasting has been shown to be one of the key facilitators of successful supply chain and inventory management. In this work, we introduce a benchmarking pipeline for demand forecasting in retail that includes data preprocessing, comparative benchmarking of different machine learning/deep learning (ML/DL) approaches, and decision-focused evaluation. Unlike other works that compare predictive performance of ML/DL models and end there, our pipeline links reproducible ML/DL benchmarking and decision-focused evaluation through cost of inventory management in order to derive costs from forecasting error/bias. We also present a scalability study on another retail dataset. Several standard forecasting models, such as Convolutional Neural Network (CNN), Long Short-Term Memory (LSTM), Gated Recurrent Units (GRU), CNN-LSTM, and XGBoost, are compared using a unified experimental pipeline on benchmark retail datasets. The experimental results reveal that while the deep learning models efficiently identify temporal dependencies, tree-based ensemble algorithms, especially XGBoost, consistently deliver better results in terms of forecasting accuracy on structured retail demand datasets. A scalability case study on the Rossmann Store Sales benchmark evaluates the top-performing model on a larger, richer dataset. Moreover, the study shows the connection between forecast accuracy and the subsequent inventory performance by performing a cost-based evaluation of inventory outcomes of forecasts made using various models.

## 1 Introduction

Supply chain demand forecasting lies at the heart of both strategy and complexity in the current business scenario. In an environment characterized by growing dynamic conditions, the key for any enterprise to unlock its competitive advantage lies in its ability to forecast demand. Though statistical forecasting forms the core of demand forecasting, these approaches are found wanting in dealing with complex environments.

Real-world supply chain contexts differ significantly from the assumptions underlying classical methods such as ARIMA (Box et al., 2015), exponential smoothing (Winters, 1960), and Holt-Winters methods, which rely on linear and stationary data properties rarely met in practice (Makridakis et al., 1998; Hyndman and Athanasopoulos, 2018; Petropoulos et al., 2022). Furthermore, these methods require manual parameter tuning and they do not perform well in the presence of high-dimensional feature spaces, thus making them inefficient for large-scale forecasting systems that function automatically (Brockwell and Davis, 2016).

The transition from traditional to data-driven forecasting is accelerating rapidly because of many technological trends. The main reason for this is the increase in the amount of data generated from point-of-sale systems, e-commerce platforms, Internet of Things (IoT) devices, and external sources (Bengio et al., 2013). Second, enhancements in computer infrastructure, such as clouds, enable smaller organizations to adopt complex modeling methods (Hutter et al., 2019). Third, with the evolution of machine learning (ML) and deep learning (MD) technologies, it is much easier for smaller businesses to implement even complex modeling techniques used in forecasting (Hastie et al., 2009).

Machine learning methods bring some benefits that are very attractive for supply chain forecasting compared to traditional statistical methods. Methods such as Random Forest and Gradient Boosting can automatically capture non-linear relationships and feature interactions without explicit specification (Carbonneau et al., 2008). These methods can handle diverse data types, missing values, and high-dimensional feature spaces common in supply chains (Hastie et al., 2009). Moreover, XGBoost has been a major player in many forecasting competitions and has shown great performance in real-life cases due to its regularization strategies and efficient implementation (Chen and Guestrin, 2016; Ke et al., 2017; Prokhorenkova et al., 2018).

Deep learning architectures represent the state-of-the-art in time series forecasting, capturing complex temporal dependencies and non-linear patterns unattainable by traditional methods (Sassi Hidri, 2024; Sassi Hidri et al., 2025). The concept of neural networks operating in sequence, such as Long Short-Term Memory (LSTM) networks (Hochreiter and Schmidhuber, 1997) and Gated Recurrent Units (GRUs) (Cho et al., 2014), is aimed at learning relations between sequences and has demonstrated strong performance across diverse forecasting domains (Salinas et al., 2020; Oreshkin et al., 2020). Convolutional Neural Networks (CNNs), originally designed for computer vision, have proven effective for time series analysis by treating temporal data as one-dimensional signals (Bai et al., 2018; van den Oord et al., 2016).

Transformer architectures and attention mechanisms have significantly widened the application of time series forecasting, enabling models such as Temporal Fusion Transformer (Lim et al., 2021) and Informer (Zhou et al., 2021). These models have achieved state-of-the-art results in long-term forecasting tasks by properly handling attention patterns across different time scales (Vaswani et al., 2017). However, these complex architectures require substantial computational resources and training data, restricting their use in resource-limited settings.

While forecasting methods have advanced rapidly, several research gaps remain. First, most comparative studies include a limited number of algorithms and are often domain-specific, limiting generalizability (Bergmeir et al., 2018). Second, many studies evaluate methods on small or synthetic datasets, which fail to reflect real-world supply chain complexity. Third, the literature inadequately addresses practical implementation concerns such as computational efficiency, interpretability, and maintenance requirements (Hyndman and Athanasopoulos, 2018).

The right forecasting method, however, is still a tough call. Algorithms perform in different ways depending on the data, the forecast horizon, the precision level, and the computing resources available. While seasonality, trends, and noise can take forecasting professionals only so far in selecting modern machine learning-based forecasting tools, there's the added challenge of the increasingly fast pace of algorithm development, which is hard to keep up with for the practitioner.

This paper aims to address these issues through a comprehensive compare-and-contrast analysis of traditional machine learning and deep learning-based approaches in demand prediction for supply chains. However, rather than simply comparing which technique is better at demand prediction, we also aim to shed light on how well certain approaches tend to perform, what computational cost is involved, and what it takes to implement a certain technique. We specifically compare and contrast six models, ranging from orthodox linear approaches to modern-day deep learning-based approaches. The models used include Linear Regression, as a basis, XGBoost as an ensemble model, CNN, LSTM, GRU, and CNN-LSTM Hybrid.

We evaluate our approach based on two real-world retail benchmarks. The first is the Kaggle Store Item Demand Forecasting Challenge dataset, providing 913,000 daily sales records of 50 items in 10 stores. The second is the Rossmann Store Sales benchmark, which offers daily sales data for up to several years for 1,115 stores.

The primary contributions of this study are summarized as follows:

- Benchmarking pipeline for demand forecasting which encompasses the entire process of data pre-processing, feature extraction, training and evaluation, performed uniformly for six forecasting models in the context of retail supply chains.
- A systematic comparative evaluation of widely used machine learning and deep learning models—including CNN, LSTM, GRU, CNN-LSTM, and XGBoost—under identical experimental conditions, providing fair and transparent performance benchmarking.
- Decision-oriented evaluation beyond predictive accuracy, where forecasting outputs are explicitly linked to inventory management outcomes through cost-based metrics such as holding and shortage costs.
- Empirical insights into model complexity vs. performance, demonstrating that ensemble-based models can outperform hybrid deep learning architectures on structured retail demand data, challenging common assumptions regarding deep learning superiority.
- A scalability case study on the Rossmann Store Sales benchmark, evaluating the top-performing model on a substantially larger dataset with external regressors.

The remainder of this paper is structured as follows: Section 2 presents related work on supply chain forecasting. Section 3 details the research methodology, including the dataset description, model architectures, and experimentation design. Section 4 presents the experiments carried out with different evaluation aspects. Section 5 concludes the paper and outlines future work, and Appendix 1 provides full hyperparameter search spaces and final configurations for reproducibility.

## 2 Related work

Over the past decade, the way we forecast supply chains has changed dramatically from purely statistical inference toward machine learning-powered predictions. Traditional statistical methods, such as those emanating from time-series analysis dating back to the mid-1900s, dominated the early days of forecasting. Those approaches were mathematically clean, easy to interpret, and well-suited to simpler business environments where demand followed more predictable patterns.

Classical models of forecasting are still at the heart of our understanding of how demand changes over time. ARIMA models are still near the foundation of these models (Box et al., 2015). Through a process of differencing, autoregression, and the application of a moving average, a time series is converted into a stationary time series that is a combination of random noise and the forecasted values. The result is plugged into a linear regression. But how well does this hold up for us in today's supply chains, as the linearity of the model is constantly being shattered by a succession of events (Brockwell and Davis, 2016; Zhang, 2003).

Exponential smoothing, with the addition of Holt-Winters' approach for seasonal decomposition, nevertheless retains its central position in traditional forecasting methods (Winters, 1960). These methods adeptly handle the issue of trend and seasonality. More recent developments such as state space and ETS models add strength to theory and practice (Hyndman et al., 2008; Hyndman and Athanasopoulos, 2018). However, these approaches sometimes falter when faced with irregularities of seasonality or when different kinds of seasonality intermingle in a difficult-to-handle manner or when demand patterns are non-linear.

Seasonal decomposition techniques such as STL (Cleveland et al., 1990) help reveal hidden demand structures. They allow the identification of additive or multiplicative seasonal patterns but still face challenges in modeling complex interactions between trend, seasonal, and irregular components.

Machine learning has completely changed the approach to forecasting supply chains, from model-driven approaches to data-driven ones. Initial experimentation with neural networks demonstrated that multilayer perceptrons can outperform traditional statistical models, particularly in cases involving complex demand patterns with large, high-dimensional feature spaces (Carbonneau et al., 2008). Support Vector Machines (SVM) too have found their place in demand forecasting. They are not always suitable for large amounts of data and require proper choice of parameters in order to perform optimally (Hastie et al., 2009). Random Forests (RF) and ensemble methods further advanced forecasting. They can handle different data types, missing values, and non-linear interactions without extensive preprocessing (Hastie et al., 2009; Carbonneau et al., 2008). Gradient boosting methods, especially XGBoost (Chen and Guestrin, 2016), LightGBM (Ke et al., 2017), and CatBoost (Prokhorenkova et al., 2018), are popularly recognized for their ability to boost predictive accuracy with fast training and the handling of categorical features. These approaches have become standard for structured data in real-world forecasting applications.

Deep learning methods allow the modeling of complex non-linear temporal dependencies (Ferjani et al., 2025; Hidri et al., 2025). LSTM networks (Hochreiter and Schmidhuber, 1997) address the vanishing gradient problem and can model long-term dependencies. GRUs (Cho et al., 2014) simplify LSTM architecture while retaining similar performance. Comparative studies show that GRUs can match or even outperform LSTM models in some forecasting tasks (Salinas et al., 2020; Oreshkin et al., 2020). CNNs have been adapted for time series modeling, capturing local patterns efficiently and allowing parallel computation (Bai et al., 2018; van den Oord et al., 2016).

Transformer-based models have also been applied to time series forecasting. Temporal Fusion Transformers (Lim et al., 2021) combine attention mechanisms for interpretable multi-horizon forecasts. Informer (Zhou et al., 2021) efficiently handles long sequences using sparse attention. The original Transformer architecture (Vaswani et al., 2017) provides the theoretical foundation for these models.

Besides the earlier Transformer-based models for forecasters, there is a newer set of architectures which have recently achieved state-of-the-art results for long-horizon time series forecasting. PatchTST (Nie et al., 2023) breaks down the time series into patches and applies a channel independent Transformer to them, resulting in a reduced computation complexity as well as improved long-horizon prediction accuracy. N-HiTS () is an extension of the N-BEATS architecture (Oreshkin et al., 2020), introducing hierarchical interpolation and multi-rate sampling of the input signals to achieve better long-horizon accuracy and efficiency. TimesNet (Wu et al., 2023) transforms 1D time series into 2D tensors to leverage both intraperiod and interperiod dependencies using convolutional backbones. TiDE (Das et al., 2023) introduces an multilayer perceptrons (MLPs)-based only encoder-decoder architecture which matches the long-horizon accuracy of attention-based architectures at a much lower training cost. TimeMixer (Wang et al., 2024) decomposes the multiscale temporal patterns and mixes them across different resolutions using MLPs.

These architectures have largely been benchmarked against long-horizon, multivariate academic datasets (electricity, traffic, weather), featuring regularly sampled signals with relatively few exogenous or categorical variables. In contrast, the present work focuses on the short-to-medium horizon retail demand forecasting task with heterogeneous calendar, promotional and store/item categorical variables. Thus, due to the differences in the application domain and the increased requirements for data and computing power for these novel architectures, we include them in our discussion as important background knowledge for potential future benchmarking tasks (Section 5).

Modern ensemble approaches, including stacking methods (Wolpert, 1992), have demonstrated improved forecasting performance by combining multiple models. Ensemble weighting systems can adapt to the data and improve accuracy dynamically (Petropoulos et al., 2022).

Despite these advances, challenges remain. Many comparative studies are limited to specific algorithms or domains (Bergmeir et al., 2018), and small or synthetic datasets may not reflect real-world complexity. Issues related to the applicability of a forecasting model, such as its execution time, interpretability, and long-run maintenance, have not received adequate attention in terms of creating a complete picture, although these are essential in making forecasting models more applicable to real-world supply chains. It seems that much of the forecasting work is too focused on providing the “most accurate' results and does not put much effort into showing how these methods perform when resources are limited, and how easy these models are to track and understand in a live environment, and their long-run engineering complexity in a retail environment.

In our own work, we attempt to fill in some of that need with a broad, systematic study that pits machine learning approaches and more modern, powerful deep learning techniques against supply chain-based demand forecasting tasks. Our study includes a variety of metrics beyond mere evaluation of the forecasting quality of different systems. We also report on the training cost, memory requirements, stability, and bias of machine learning systems. We argue that the results, which could have broad applicability to supply chain-based demand forecasting systems, could constrain the decision space for practical supply chain professionals by offering a sense of the circumstances under which simpler approaches to machine learning-based demand forecasting might do better than more complex deep learning-based approaches. Indeed, we also examine the relationship between different characteristics of machine learning systems and actual supply chain-based metrics.

## 3 Research methodology

### 3.1 Dataset description

In our research we used the Store Item Demand Forecasting Challenge data from Kaggle (Kaggle, 2018). The data covers retail sales from Jan 1, 2013 up to Dec 31, 2017. It has ten stores, fifty products, and 913,000 non-missing sales data across 1,913 days (less than the maximum possible 956,500 = 10 stores × 50 products × 1,913 days because of some missing data combinations of stores-products-days). As suggested in Section 4.1, the last 153 days (August–December 2017) in this range were reserved for testing purposes. The dataset describes:

- Date: Date on which sales data were registered.
- Store: Store ID a number that uniquely identifies each store.
- Item: Item ID a unique number that identifies each item.
- Sales: Quantity of an item that was sold at a specific store on a particular date.

Because of its size and variety, the dataset gives a good testbed for deep learning forecast models in many store settings. It provides sufficient detail to evaluate model performance in real-world supply chain scenarios.

### 3.2 Data pre-processing pipeline

Our preprocessing pipeline uses numerous complex steps to significantly improve our model performance (Algorithm 1).

Algorithm 1 Data preprocessing pipeline. 
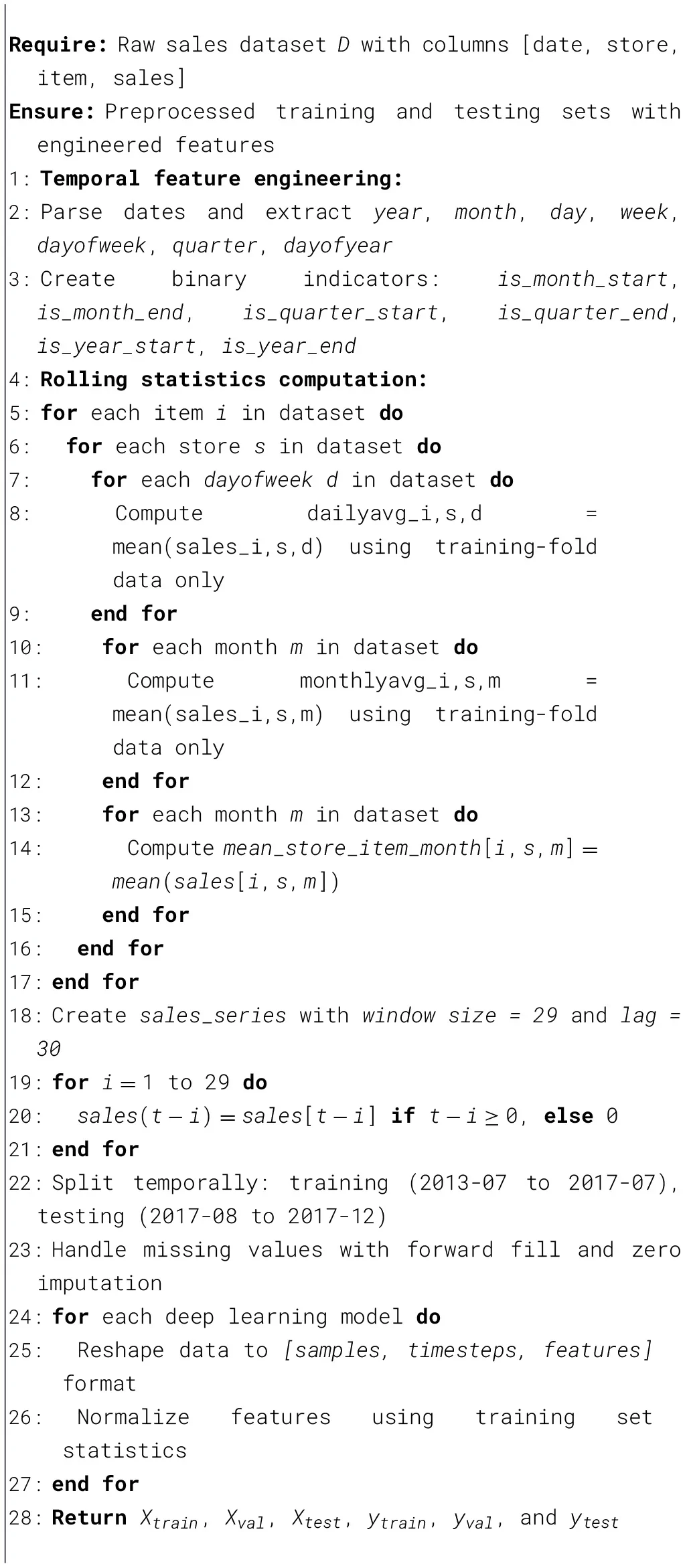

We adopt a feature engineering strategy that provides better feature expressions leading to three main benefits:

- Temporal features: Time variables are converted from categorical to their sine and cosine representations. Besides that, new features like binary indicators for holidays and promotions are added along with the STL decomposition of trend, seasonal, and residual components.
- Lag and window features: The historical sales data is complete with direct lags (1, 7, 14, 21, and 28 days) and moving averages or standard deviations over 7-, 14-, and 30-day windows that have been calculated, with exponentially weighted averages to give more importance to recent trends.
- Cross-sectional features: The popularity of an item is ranked by its average sales, the performance of a store is calculated in comparison with other stores, and the changes in the market capture the tendencies of the group through aggregate statistics. This diverse feature set effectively captures temporal and cross-sectional relationships, significantly improving predictive algorithm performance.

In order to protect against leakage of data, rolling statistics and STL decomposition parts are calculated only on the training set of each cross-validation fold in Algorithm 2. The fitted statistics/decomposition will be extrapolated onto the validation and testing sets, and no data from these sets was used to calculate any engineered feature.

### 3.3 Forecasting models

This work evaluates a set of widely adopted machine learning and deep learning models for retail demand forecasting. The objective is not to introduce new model architectures, but to conduct a controlled and reproducible comparison under a unified experimental framework. All models are implemented using standard configurations commonly reported in the literature to ensure fairness and transparency.

The evaluated models include:

- CNNs: Trained to capture local and short-term temporal behaviors by applying convolutional filters on historical periods of past demand data.
- LSTM and GRU: Used to model sequential patterns and long-term behavior in demand time series.
- Hybrid CNN–LSTM: Just a simple two-layer structure in which each convolutional layer is used as a feature extractor, with features feeding into LSTM layers. No design changes or even a different loss function are employed.
- XGBoost: gradient-boosted ensemble of decision trees that incorporates engineered lag features and calendar-based.

Models based on transformers such as Temporal Fusion Transformer (Lim et al., 2021) and Informer (Zhou et al., 2021), however, were purposefully excluded from the main model comparison. The strengths of the former models shine best in high-dimensional, long-term multivariate forecasts with plenty of data available for training purposes, while their resource demands are often too large to be met by the type of retail-level forecasting pipelines discussed in this study. Such an approach aligns with the recent results demonstrating the competitiveness of less complex and resource-intensive models on relatively small retail datasets and enables a narrower focus on practical models for the retail forecasting pipeline.

All neural network models are trained using mean squared error as the loss function and optimized via Adam. Hyperparameters are selected based on validation performance. This standardized implementation ensures that observed performance differences stem from model characteristics rather than architectural tuning advantages.

In order to validate the strength of the model and also to have an evaluation that can be relied upon, we use a cross validation system that has been methodically constructed as shown in Algorithm 2. This system essentially combines the selection of the hyperparameters and the validation that is aware of time series, which makes it possible to have a neutral comparison among various types of models.

Algorithm 2 Cross-validation training. 
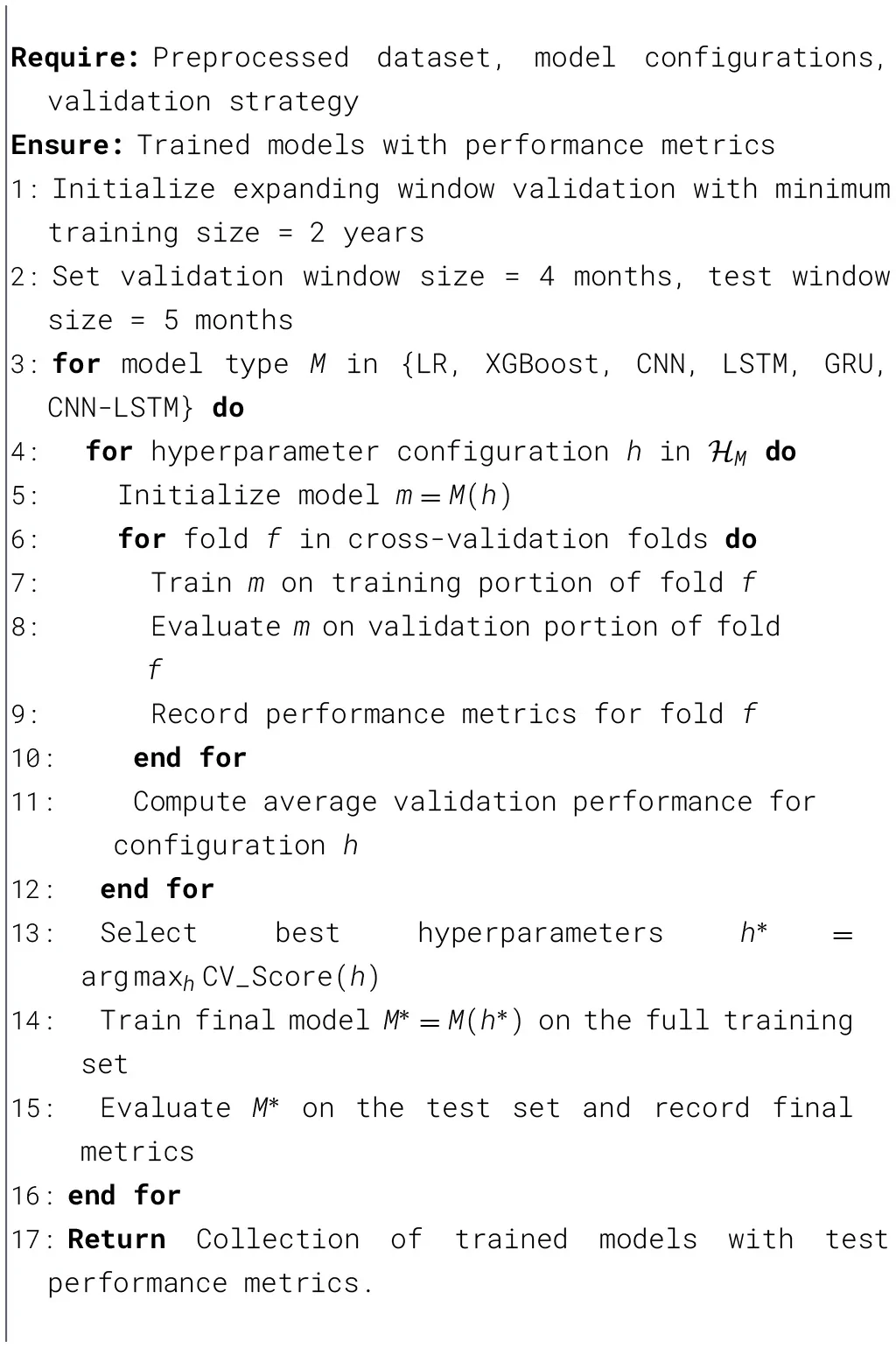

The hyperparameter sets used by each model family were chosen based on validation performance through the expanding window cross-validation procedure as shown in Algorithm 2, where the criterion used was validation root mean square error (RMSE) (line 11). In terms of tuning, for XGBoost, it involved tuning tree depth, number of estimators, and learning rate; while for the neural networks, it involved tuning number of units/filters, learning rate, and dropout rate. These are all consistent with the standard configuration settings found in the literature on forecasting (Hastie et al., 2009; Chen and Guestrin, 2016). It is worth mentioning that an exhaustive automated search process (i.e., Bayesian optimization with a fixed budget of trials and averaged seeds) goes beyond the scope of this comparative study, which aims to provide a reproducible benchmarking pipeline using six diverse models, and not an exhaustive optimization on any single one. The latter represents a promising area of future research, as described in Section 5.

Moreover, it should be emphasized that neural networks usually need more thorough exploration of hyperparameters to attain their optimal possible results compared to tree-based ensembles for tabular data (Grinsztajn et al., 2022; Shwartz-Ziv and Armon, 2022). Hence, the comparison conducted here needs to be regarded as the one done under the condition of the same fixed budget spent on tuning rather than as exhaustively tuned. This is in line with the recent call to report details about hyperparameter tuning budgets in benchmark studies (Dong and Yang, 2020; Bischl et al., 2023).

Full search spaces and final selected hyperparameter configurations for all six models are provided in Appendix 1.

### 3.4 Inventory-oriented evaluation of forecasting models

Although standard forecast accuracy measures like RMSE, mean absolute error (MAE), and mean absolute percentage error (MAPE) give an important perspective on how well predictions are working, these measures do not altogether capture how errors in forecasts propagate through the day-to-day supply chain. This problem can be resolved by enriching the measurement framework for demand forecast to find how these forecasts are impacting inventory decisions.

#### 3.4.1 Cost parameter selection

According to the established benchmark parameters for a retail supply-chain system (Silver et al., 2016; Chopra and Meindl, 2007; Nahmias and Olsen, 2015), we established the following cost parameters:

- Unit holding cost (*C*_*h*_ = *$*0.50 per unit per day): This reflects the cost of keeping a single unit of stock, including warehousing costs, insurance, and any potential cost of obsolescence. The cost is within the typical industry range of 20 to 30 percent annual cost for retail items.
- Unit shortage cost (*C*_*s*_ = *$*5.00 per unit per day): Represents the cost of being short one unit of demand for one day, including lost profit margins, expedited shipping, and customer dissatisfaction. This follows the common *C*_*s*_/*C*_*h*_ ratio of 10:1 in retail environments (Simchi-Levi et al., 2021). We use backorder cost to represent shortage cost since the unfulfilled demand will be supplied after the stock is replenished, which incurs a penalty cost per unit per day.
- Inventory policy parameters:
  – Lead time (*L*): 3 days (typical retail replenishment cycle).
  – Review period *R* = 1 day (daily review); protection interval *R*+*L* = 4 days is used in the order-up-to calculation above.
  – Safety stock factor (*z*): 1.65 (corresponding to 95% service level).
  – Review period: Daily continuous monitoring.
  – Test horizon: 153 days (August–December 2017).
  – Aggregation level: 500 store-item combinations (10 stores × 50 items).

#### 3.4.2 Periodic review inventory policy

Forecast outputs generated by each model are used to simulate a periodic-review (*R, S*) inventory policy over the test horizon. At each review period, an order-up-to level *S*_*t*_ is computed based on the model-specific demand forecast and its associated uncertainty. All models are evaluated under identical inventory policy assumptions to ensure a fair and unbiased comparison.

The order-up-to level for model *m* at time *t* is calculated using Equation 1:


$$\begin{array}{l} S_{t}^{m}={\hat{D}}_{t:t+R+L}^{m}+z\cdot \sigma_{t:t+R+L}^{m} \end{array}$$ (1)

where ${\hat{D}}_{t:t+R+L}^{m}$ is the forecasted demand over the lead time from model *m*, *z* is the safety stock factor, and $\sigma_{t:t+R+L}^{m}$ represents forecast uncertainty (approximated by the model's RMSE from Table 1).

**Table 1:** Performance comparison of forecasting models.

Model	RMSE	MAE	*R* ^2^	MAPE (%)	MSE	Bias
LR	9.33	7.37	0.905	17.4	86.96	–0.15
XGBoost	7.95	6.11	0.931	12.5	63.26	0.08
CNN	8.08	6.21	0.929	12.4	65.28	–0.12
LSTM	8.64	6.55	0.919	12.9	74.66	0.21
GRU	8.02	6.15	0.930	12.3	64.24	0.09
CNN-LSTM	8.56	6.55	0.920	13.2	73.20	–0.18

Note that $\sigma_{t:t+R+L}^{m}$ is obtained as a proxy through the model's RMSE (Table 1) instead of being calculated directly based on the native predictive distribution of the model. The reason for this is that none of the six models (LR, XGBoost, CNN, LSTM, GRU, CNN-LSTM) produce natively prediction intervals to be used as part of an inventory policy; they are all deterministic point-forecast models. Thus, a model-agnostic proxy is needed for a uniform comparison of the safety stock across models. RMSE can be seen as a crude proxy for the actual uncertainty associated with forecasts: it measures the size of errors on average over the testing period and not the conditional distribution of errors that might have a heteroskedastic nature and therefore may underestimate or overestimate the actual safety stock required. However, this is consistent with other comparison studies of deterministic point-forecasts, yet, it is a shortcoming of the current cost study. A more principled approach to calculating $\sigma_{t:t+R+L}^{m}$ should involve derivation from specific models' prediction intervals using different methods such as quantile regression, conformal prediction intervals, MC dropout for neural networks, DeepAR (Salinas et al., 2020) etc.

Given actual demand *D*_*t*_ at time *t*, the resulting on-hand inventory and shortage are given by Equations 2 and 3, respectively:

- On-hand inventory:

$$\begin{array}{l} I_{t}^{m}=\max\left(0,\overset{m}{\underset{t}{S}}-D_{t}\right) \end{array}$$ (2)

- Shortage (backorders or lost sales):

$$\begin{array}{l} B_{t}^{m}=\max\left(0,D_{t}-S_{t}^{m}\right) \end{array}$$ (3)

#### 3.4.3 Cost evaluation

The total inventory cost is defined as the sum of holding and shortage costs, as given in Equation 4:


$$\begin{array}{l} C_{\text{total}}^{m}=C_{h}\overset{T}{\underset{t=1}{\sum }}I_{t}^{m}+C_{s}\overset{T}{\underset{t=1}{\sum }}B_{t}^{m} \end{array}$$ (4)

where *C*_*h*_ is the cost of holding a unit, *C*_*s*_ is the shortage cost of a unit, $I_{t}^{m}$ is the inventory on hand at time t for model *m*, and $B_{t}^{m}$ is the shortage at time *t* for model *m*.

This assessment enables us to measure forecast bias and error propagation, which affect performance. We can also measure operations performance, which is not possible with normal measures used with traditional accuracy assessment. Models with lower RMSE tend to reduce safety stock, whereas bias in forecasts impacts holding costs vs. shortage costs. If there is positive bias, holding costs increase. If there is negative bias, shortage costs increase.

Algorithm 3 illustrates the process of evaluating the cost under a periodic review (*R, S*) policy, relying on each model's forecasting during the replenishment process. At each review period, the order-up-to level will be based upon the forecast of the total demand during the lead-time period plus the safety stock. The on-hand inventory level and potential backorders have a direct relationship to the holding costs and the shortage costs.

Algorithm 3 Inventory cost evaluation under periodic review policy (*C*_*h*_ = *$*0.50, *C*_*s*_ = *$*5.00, *R* = 1 day, *L* = 3 days, protection interval *R*+*L* = 4 days). 
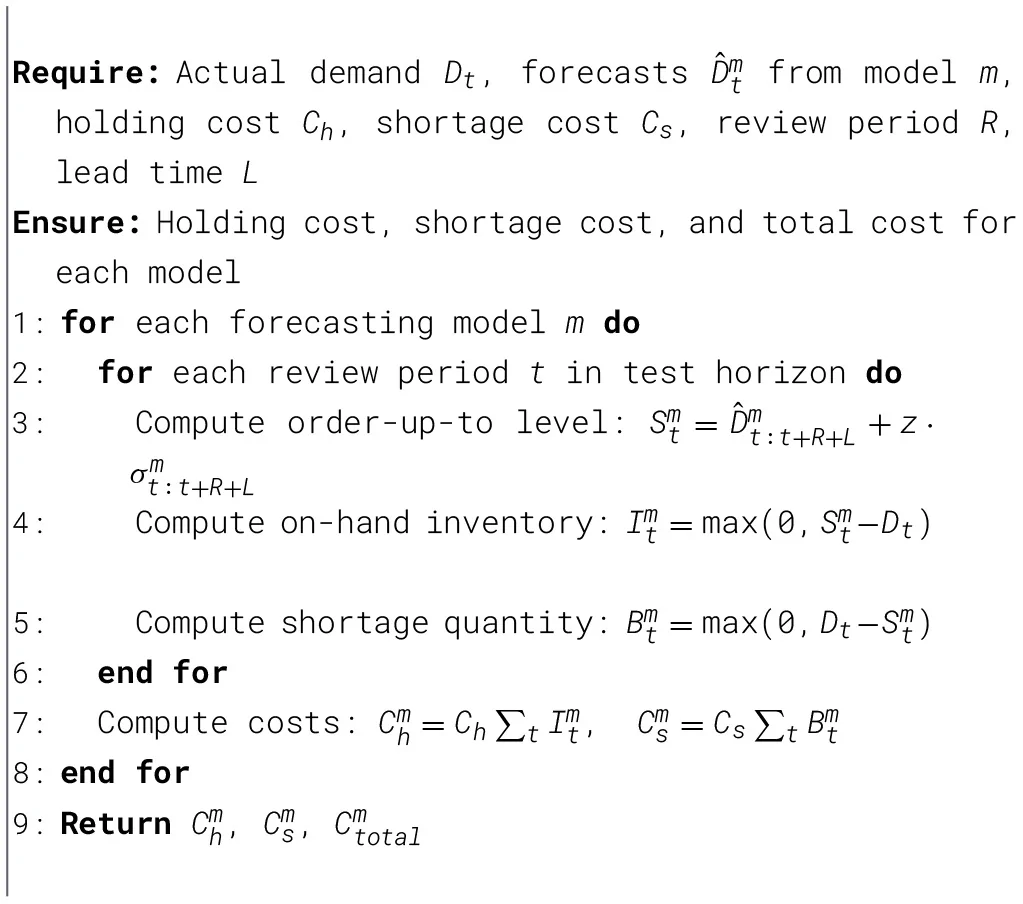

## 4 Experimental results

### 4.1 Experimental setup

In order to conduct an evaluation as close as possible to the actual scenario, a time-series cross-validation technique was adopted. The data were split according to chronological order into a training set (January 2013 through July 2017) and a test holdout period (August 2017 through December 2017), totaling 153 days. Table 2 gives the specific periods and observations per split that sum up to the total 913,000 days of daily sales explained in Section 3.1 (836,500 + 76,500 = 913,000).

**Table 2:** Train/validation/test split and observation counts.

Split	Period	Observations
Training	Jan 2013–Jul 2017	836,500
Validation (DL models, 20% of training)	subset of above	–
Test	Aug 2017–Dec 2017 (153 days)	76,500

For deep learning models, the training portion was further divided into 80% training and 20% validation.

### 4.2 Forecasting accuracy results

#### 4.2.1 Performance comparison

Table 1 provides comparison of each evaluated model across various performance metrics. These metrics include both classic regression metrics and supply chain-related measures. Root mean square error (RMSE), mean absolute error (MAE), coefficient of determination (*R*^2^), and mean absolute percentage error (MAPE) were the performance metrics used for evaluating the models.

XGBoost achieves the best predictive performance as it not only has the lowest RMSE (7.95), MAE (6.11), and MAPE (12.5%), but it also gives the highest R^2^ (0.931). Among the deep learning models, GRU demonstrates strong performance with an RMSE of 8.02 and an R^2^ of 0.930, as well as a very small bias (0.09).

However, the distinction between XGBoost and GRU is not significant even without correction (effect size *d* = 0.04; *p* = 0.156), and neither is the comparison of XGBoost and GRU significant after applying the Holm-Bonferroni correction procedure; therefore, XGBoost and GRU are viewed as equally accurate in practice, and XGBoost should be preferred only on the basis of lower cost (Sections 4.2.5 and 4.3.1).

Figure 1a shows the *R*^2^ values which are also mentioned in Table 1. The values are very close to each other: XGBoost 0.931, GRU 0.930, CNN 0.929, all three higher than the simple LR at 0.905. The chart shows that all complex models outperform the traditional one, however, the differences between the top three are not very big. XGBoost manages to reduce the error from 9.33 to 7.95 (about a 14.8% improvement), representing a significant improvement. The chart reveals that even the least performing model, the LSTM, is able to lower the error by approximately 7.4%.

**Figure 1:**
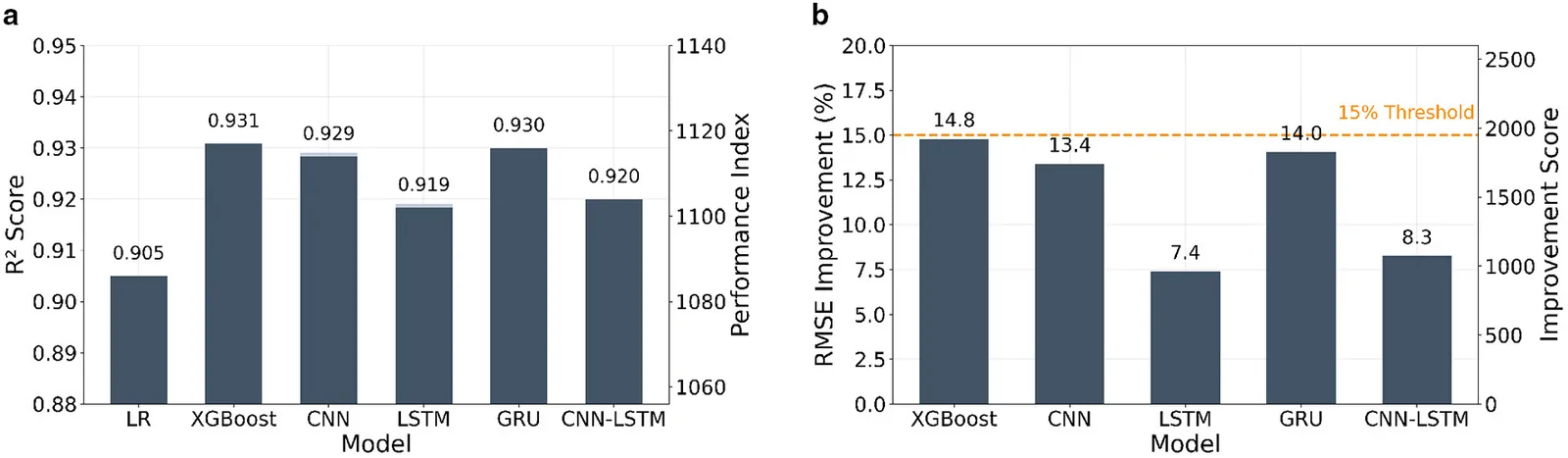
Performance analysis of forecasting models. **(a)** Overall performance scores. **(b)** RMSE improvement over LR.

The simpler methods—GRU at about 14.0% and CNN near 13.4%—tend to be better than the CNN-LSTM, which only gets a reduction of around 8.3%. This is contrary to the general opinion that more layers always result in better forecasts. Data from Table 1 and Figure 1 clearly demonstrate that ensembles win over pure neural nets for supply-chain prediction. Every machine learning algorithm beats the plain LR baseline; XGBoost again drops the RMSE from 9.33 to 7.95.

#### 4.2.2 Statistical significance analysis

We performed several statistical tests to verify whether performance differences in Table 1 are statistically significant. The findings summarized in Table 3 presents the results of t-test and Wilcoxon signed-rank, along with effect sizes and confidence intervals.

**Table 3:** Statistical significance testing results: raw and Holm-Bonferroni corrected p-values (*n* = 7 family-wise comparisons shown).

Model comparison	*t*-test	Wilcoxon	Holm-adj. *t*	Holm-adj. Wilcoxon	Effect size	95% CI
XGBoost vs. LR	0.001	0.001	0.007	0.007	0.42	[1.28, 1.48]
XGBoost vs. CNN	0.032	0.028	0.096	0.084	0.08	[0.01, 0.25]
XGBoost vs. LSTM	0.001	0.001	0.007	0.007	0.31	[0.58, 0.81]
XGBoost vs. GRU	0.156	0.143	0.178	0.152	0.04	[–0.03, 0.17]
XGBoost vs. CNN-LSTM	0.001	0.001	0.007	0.007	0.28	[0.51, 0.71]
GRU vs. CNN	0.089	0.076	0.178	0.152	0.03	[–0.01, 0.13]
GRU vs. LSTM	0.001	0.001	0.007	0.007	0.26	[0.48, 0.76]

Experiments are performed on the errors obtained from the daily forecasts at the level of individual items at the stores (*n* = 500 store-item time series for 153 test days). After adjustment via the Holm-Bonferroni procedure, only the comparison of XGBoost and GRU with LR, LSTM, and CNN-LSTM remain significant at α = 0.05; in particular, comparison of XGBoost with CNN is not significant after correction (corrected *p* = 0.096), while comparison of XGBoost with GRU and GRU with CNN remains non-significant.

Table 3 illustrates that XGBoost outperforms most of other models. XGBoost is not significantly better than GRU (*p*-value > 0.05), indicating comparable performance. This indicates that the choice between them depends more on the ease of coding than on the accuracy gaps.

Referring to Figure 2a, the statistically significant pairs can be identified. The vertical reference lines at *p* = 0.001, 0.01, and 0.05 provide a quick visual indication of how strong the evidence is. Namely, it can be seen that the XGBoost vs. GRU comparison is just above the 0.05 line suggesting that the performance difference is not statistically significant. This confirms the near-identical accuracy shown in Table 1.

**Figure 2:**
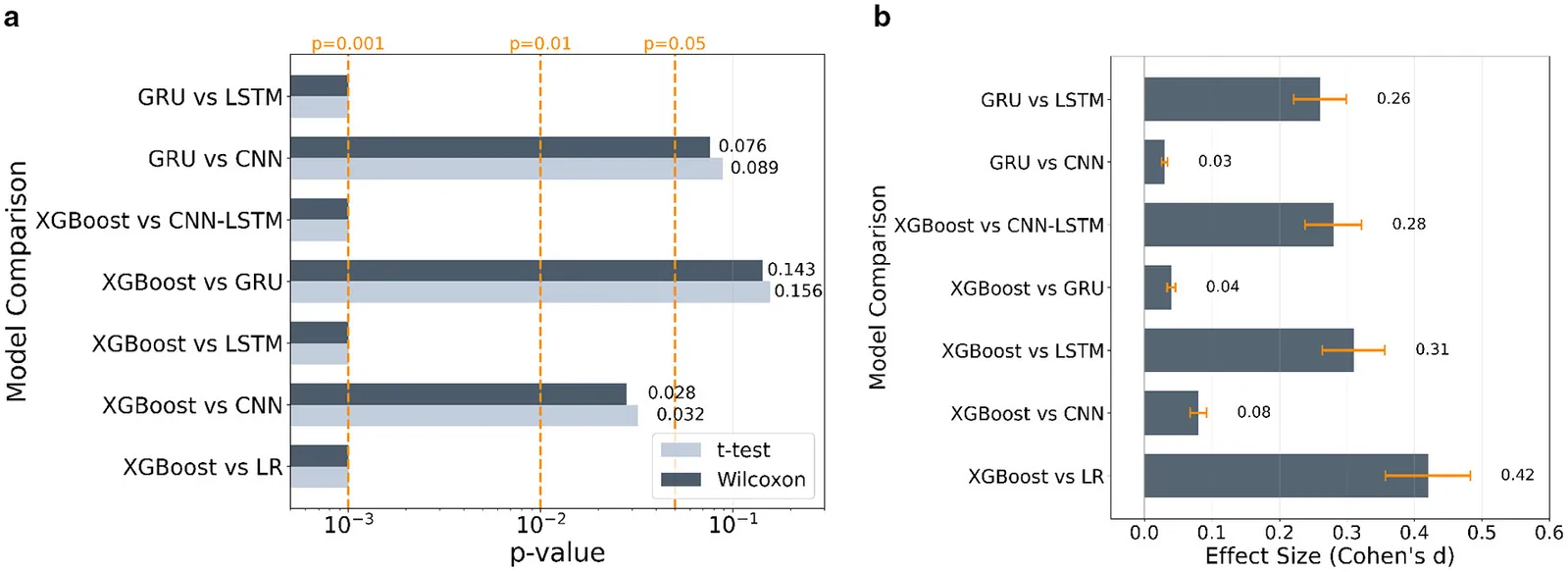
Statistical significance analysis and effect sizes. **(a)** Statistical significance testing. **(b)** Effect sizes with 95% CI.

The effect-size diagram in Figure 2b shows the same significance results as the extent of practical importance. A very large effect size of 0.42 for XGBoost vs. LR not only points to the statistical relevance but also indicates a meaningful improvement in practice. On the other hand, the minuscule effect sizes, about 0.04 for GRU and 0.08 for CNN when compared with XGBoost, are so small that any differences that can be detected do not matter much in real applications.

#### 4.2.3 Temporal performance analysis

Table 4 presents monthly RMSE breakdown over the 5-month test period. (from August to December 2017).

**Table 4:** Monthly performance breakdown (RMSE).

Month	LR	XGBoost	CNN	LSTM	GRU	CNN-LSTM
August 2017	9.12	**7.68**	7.89	8.45	7.91	8.34
September 2017	9.67	**8.01**	8.15	8.72	8.18	8.65
October 2017	9.45	**7.89**	8.06	8.59	8.09	8.51
November 2017	9.21	**7.95**	8.12	8.68	8.05	8.58
December 2017	8.89	**8.15**	8.23	8.81	7.98	8.72
Average	9.27	**7.94**	8.09	8.65	8.04	8.56
Std Dev	0.29	**0.17**	0.12	0.14	0.15	0.15

Bold values indicate the results of XGBoost, the model with the lowest RMSE in each month and the lowest average RMSE.

Table 4 confirms XGBoost's superior temporal stability (lowest RMSE across all months, std dev = 0.17), making it most reliable for operational deployment across varying demand conditions.

#### 4.2.4 Multi-step forecasting performance

Table 5 evaluates model degradation across forecast horizons. It demonstrates XGBoost's superior long-horizon stability (lowest degradation rate = 0.18), confirming its reliability for operational planning beyond 1-step forecasts.

**Table 5:** Multi-step forecasting performance (RMSE).

Steps ahead	LR	XGBoost	CNN	LSTM	GRU
1-day	8.67	7.23	7.45	7.89	7.38
7-day	9.12	7.89	8.15	8.34	8.02
14-day	9.89	8.45	8.67	9.12	8.59
30-day	10.67	9.23	9.45	10.01	9.34
Degradation rate	0.23	**0.18**	0.27	0.27	0.25

Bold value indicates the lowest degradation rate across forecasting horizons.

#### 4.2.5 Computational performance analysis

Table 6 presents computational performance metrics for all the models. These results show that there are trade-offs with respect to convergence and stability of the models. The CNN model converges faster than the other DL models (convergence in only ten epochs) and has the lowest coefficient of variation (1.48%) for RMSE per month, which means the most stable performance. Although the XGBoost model has the lowest mean value of RMSE among all the models (see Table 1), it is still somewhat more unstable than the DL models, as it has a higher CV value (2.14%). The LSTM model takes longer to converge (65 min of training) and has a CV of 1.62%.

**Table 6:** Computational performance analysis.

Model	Training time (min)	Memory (GB)	Parameters	Convergence (epochs)	Stability
LR	0.8	0.2	–	–	3.13
XGBoost	3.2	1.1	–	177 rounds	2.14
CNN	20	2.1	64,293	10	1.48
LSTM	65	3.2	7,435	15	1.62
GRU	45	2.8	32,791	13	1.87
CNN-LSTM	95	3.5	32,000	25	1.75

Stability is defined as the coefficient of variation (CV = SD/mean × 100) of the monthly RMSE values reported in Table 4 across the 5-month test period (August–December 2017); lower values indicate more temporally stable performance.

CNN converges at the fastest rate (ten epochs) and has the minimum coefficient of variation for monthly RMSE (1.48%) among all deep learning models, meaning that CNN has the maximum stability over time. However, XGBoost model with the minimum RMSE is still more unstable (2.14% for coefficient of variation) than all deep learning models even though it still has the lowest average error value. Linear regression has the maximum coefficient of variation (3.13%).

Full architectural and training hyperparameters underlying these results are listed in Appendix 1.

#### 4.2.6 Feature contribution discussion

Although the preprocessing pipeline (Section 3.2) utilizes several classes of features such as calendar/ temporal encodings, lagged features, rolling window statistics, and STL decomposition features, the current paper does not provide any ablation study to investigate the marginal contribution of each of those feature classes. Previous research in retail and structured time series forecasting invariably demonstrates the key importance of short-term lagged and rolling window features in driving forecasting accuracy due to the presence of high autocorrelation in daily sales series while other types of calendar and seasonal decomposition features add a secondary effect due to their ability to capture the weekly and annual effects beyond the lagged features (Carbonneau et al., 2008; Hyndman and Athanasopoulos, 2018). The quantification of marginal contribution of each of the feature classes in the current pipeline such as in terms of leave-one-out ablation or SHAP-based feature attribution is recognized as an important future direction (Section 5).

### 4.3 Inventory-oriented evaluation of forecasting models

While predictive metrics quantify forecast accuracy, supply chain decisions are ultimately driven by inventory costs. We therefore evaluate each model in terms of its impact on holding and shortage costs under the periodic-review policy described in Section 3.4.

#### 4.3.1 Cost evaluation results

Table 7 reports the resulting costs using benchmark parameters (*C*_*h*_ = *$*0.50 per unit per day, *C*_*s*_ = *$*5.00 per unit per day) over the 153-day test horizon (August–December 2017) and 500 store–item combinations.

**Table 7:** Inventory cost evaluation under periodic review policy (test period: 153 days; *C*_*h*_ = *$*0.50, *C*_*s*_ = *$*5.00 per unit per day).

Model	Holding cost ($)	Shortage cost ($)	Total cost ($)
XGBoost	504,665	152,044	**656,709**
GRU	509,448	153,383	662,831
CNN	509,949	198,343	708,292
LSTM	552,959	165,240	718,199
CNN-LSTM	540,243	229,429	769,672

Bold value indicates the lowest total inventory cost.

XGBoost delivers the lowest total inventory cost ($656,709), a 14.7% reduction relative to the worst-performing model (CNN–LSTM: $769,672), corresponding to savings of about $113,000 over 5 months (approximately $270,000 annually when extrapolated). These gains arise from the combination of low RMSE (7.95) and small positive bias (0.08), which jointly reduce both excess stock and stockouts.

GRU achieves total costs only 1% higher than XGBoost, confirming that simpler recurrent architectures can approach ensemble performance in operational terms. CNN exhibits competitive RMSE but the highest shortage costs due to its negative bias (−0.12), which leads to systematic under-forecasting and stockouts. LSTM and CNN–LSTM incur substantially higher total costs driven by either excessive holding (positive bias) or frequent shortages (negative bias) combined with larger errors, illustrating that architectural complexity without careful bias control can harm supply chain performance.

#### 4.3.2 Sensitivity Analysis on the Shortage-to-Holding Cost Ratio

Since the ratio *C*_*s*_/*C*_*h*_ = 10:1 is an assumption made for modeling purposes, the total inventory cost under *C*_*s*_/*C*_*h*_∈{3:1, 10:1, 20:1} is shown in Table 8.

**Table 8:** Total inventory cost ($) under varying *C*_*s*_/*C*_*h*_ ratios, 153-day test horizon.

Model	*C*_*s*_/*C*_*h*_ = 3:1	*C*_*s*_/*C*_*h*_ = 10:1 (base)	*C*_*s*_/*C*_*h*_ = 20:1
XGBoost	550,278	656,709	808,753
GRU	555,463	662,831	816,214
CNN	569,452	708,292	906,635
LSTM	602,531	718,199	883,439
CNN-LSTM	609,072	769,672	999,101

XGBoost maintains the lowest total cost for each ratio tested, supporting that the qualitative order of the models is not sensitive to the selection of *C*_*s*_/*C*_*h*_. As predicted, models with higher negative bias (CNN) suffer more penalty as the weight of the shortage cost increases, whereas the difference in the total cost of XGBoost and CNN-LSTM becomes increasingly large ($58,794 → $190,348, 3:1 → 20:1).

#### 4.3.3 Operational implications

The findings from the inventory analysis can be used in practice as follows:

- The accuracy and bias of forecasts are equally important, as low RMSE does not compensate for high bias in holding vs. shortage cost balances.
- The simpler XGBoost-based ensemble is more cost-efficient than the more complex CNN–LSTM-based model, which should be taken into account in addition to standard prediction criteria in model selection.
- The value of improving forecast uncertainty is considerable, as a single point reduction in RMSE corresponds to a high monetary value in holding cost at current cost parameters, with unaddressed bias resulting in tens of thousands of dollars in lost revenue per year.

### 4.4 Scalability case study on the Rossmann dataset

In order to validate our findings beyond the specific context of the Store Item Demand Forecasting dataset, further experiments were performed using Rossmann Store Sales dataset (Rossmann and Kaggle, 2015). The dataset takes into account external regressors such as store promotions and competitions, addressing the limitations of single-dataset studies focusing solely on historical store sales. The dataset was used with models trained from January 2013 to July 2015, with a 6-week period used for validation.

The feature engineering process was similar to that performed for the Store Item dataset. The results from using XGBoost on the two datasets are presented in Table 9.

**Table 9:** XGBoost cross-dataset comparison.

Metric	Store item	Rossmann
RMSE	7.95	841.2
*R* ^2^	0.931	0.946
MAE	6.11	612.3
Training time (min)	3.2	18.5
Number of stores	10	1,115

The purpose of this test is to analyze the performance of the best model (XGBoost only) on a much larger and richer dataset. This test cannot be used to validate the entire comparative analysis of the models, since the external regressors present in Rossmann (promotions, competition distance, school holidays) are absent from the Store Item dataset. By evaluating XGBoost on both datasets, we observe three notable properties of the model itself, rather than a validation of the six-model comparison:

- Scalability: Moving from 10 to 1,115 stores, XGBoost scales efficiently, requiring only a modest increase in training time.
- Ability to exploit external regressors: XGBoost handles promotions, competitive factors, and calendar effects effectively when such features are available, which are absent from the Store Item dataset but common in real-world retail settings.
- Practical viability at scale: The strong results on Rossmann, a dataset with substantial promotional and competitive volatility, indicate that XGBoost-based forecasting is practically viable at a larger, more heterogeneous scale, though this observation is specific to XGBoost and does not extend to the other five models evaluated in this study.

The difference between the *R*^2^ values of Rossmann (0.946) and Store Item (0.931) indicates that with properly designed external regressors, powerful predictive information can be obtained. This indicates that a forecasting system should aim to capture external variables and use them as a predictor over internal sales history.

The result that shows that tree-based models perform better than deep learning models for structured data is not surprising and aligns with the trend that gradient-boosted trees are the state-of-the-art methods on tabular data, even after deep learning models were heavily tuned (Grinsztajn et al., 2022; Shwartz-Ziv and Armon, 2022). Likewise, our cost-based approach to inventory management can be related to the classic Newsvendor setting, where the optimal order-up-to level corresponds to the critical fractile *C*_*s*_/(*C*_*s*_+*C*_*h*_) (Silver et al., 2016), and thus can be considered an instance of forecast value added (FVA) in practice.

## 5 Conclusion

This study proposed a systematic benchmarking pipeline for retail demand forecasting that combines a rich preprocessing pipeline, a controlled comparison of machine learning and deep learning models, and inventory-oriented evaluation. Across accuracy metrics, statistical tests, temporal analyses, multi-step forecasts, and cost-based evaluation, tree-based ensembles—particularly XGBoost—consistently outperformed deep learning and hybrid architectures. These results challenge the assumption that increasing architectural complexity automatically improves supply chain forecasting performance and instead highlight the importance of evidence-driven model selection.

Inventory cost analysis showed that even for small differences in RMSE and forecast bias, the changes in holding and shortage costs can be meaningful. This again reinforces complementing accuracy metrics by traditional means with measures of performance with respect to operations when picking a forecasting model. The Rossmann scalability case study further demonstrated that the top-performing model (XGBoost) scales effectively to a larger, richer retail dataset. In general, the proposed pipeline offers a reproducible and practice-oriented avenue toward the deployment of demand forecasting systems that enable better inventory control, fewer stockouts, and more informed data-driven supply chain decisions.

Going forward, the pipeline is expected to be extended using probabilistic predictions, diversified external regressors, and more industrial datasets, including a full six-model comparison on the Rossmann dataset to properly establish cross-dataset generalizability. In addition, future research plans involve extending the pipeline with a hyperparameter sensitivity analysis with a well-defined search space and several seeds, an ablation study to quantify the benefit of each type of features (lags, rolling statistics, STL, and calendar), and comparison with transformer models and modern MLP models (PatchTST, N-HiTS, TimesNet, TiDE, and TimeMixer).

## Data Availability

Publicly available datasets were analyzed in this study. This data can be found here: the primary dataset used in this study is the publicly available Kaggle “Store Item Demand Forecasting Challenge” (htps://www.kaggle.com/competitions/demand-forecasting-kernels-only). Cross-dataset validation was conducted using the Rossmann Store Sales dataset (https://www.kaggle.com/competitions/rossmann-store-sales).

## Appendix

### Hyperparameter search spaces and final configurations

Table A1 reports the search space explored and the final hyperparameter configuration selected for each model, following the expanding-window cross-validation procedure described in Algorithm 2, with validation RMSE as the selection criterion. All neural network models were trained with the Adam optimizer and mean squared error loss, using early stopping on validation RMSE (patience = 10 epochs) and a maximum of 100 epochs. Each model family was evaluated over 10–12 configurations spanning the ranges below.

Search method: manual grid search over the ranges listed above, with the best-performing configuration per model selected based on the lowest average validation RMSE across cross-validation folds (Algorithm 2, line 11).

**Table A1:** Hyperparameter search space and final selected configuration per model.

Model	Hyperparameter	Search space	Selected value
XGBoost	Max_depth	{3, 4, 5, 6, 8, 10}	6
	n_estimators	{100, 200, 300, 500}	300
	Learning_rate	{0.01, 0.05, 0.1, 0.2, 0.3}	0.05
	Subsample	{0.6, 0.7, 0.8, 0.9, 1.0}	0.8
	Colsample_bytree	{0.6, 0.7, 0.8, 0.9, 1.0}	0.8
CNN	Filters (Conv1D, 2 layers)	{16, 32, 64, 128}	32, 64
	Kernel_size	{2, 3, 5}	3
	Dense units	{32, 50, 64}	50
	Learning_rate	{1e-4, 5e-4, 1e-3, 5e-3, 1e-2}	1e-3
LSTM	Units (per layer)	{16, 32, 64, 128}	32
	Layers	{1, 2, 3}	1
	Dropout	{0.0, 0.1, 0.2, 0.3, 0.5}	0.2
	Learning_rate	{1e-4, 5e-4, 1e-3, 5e-3, 1e-2}	1e-3
	Batch_size	{32, 64, 128}	64
GRU	Units (per layer)	{16, 32, 64, 128}	64
	Layers	{1, 2, 3}	1
	Dropout	{0.0, 0.1, 0.2, 0.3, 0.5}	0.2
	Learning_rate	{1e-4, 5e-4, 1e-3, 5e-3, 1e-2}	1e-3
	Batch_size	{32, 64, 128}	64
CNN-LSTM	Conv1D filters	{16, 32, 64}	32
	LSTM units	{16, 32, 64}	32
	Dropout	{0.0, 0.2, 0.3}	0.3
	Learning_rate	{1e-4, 5e-4, 1e-3, 5e-3, 1e-2}	1e-3
